# Controlled prospective study on ultrasound simulation training in fetal echocardiography: FESIM II

**DOI:** 10.1007/s00404-023-07133-2

**Published:** 2023-07-16

**Authors:** Paul Janzing, Nasenien Nourkami-Tutdibi, Erol Tutdibi, Paula Freundt, Thomas von Ostrowski, Martin Langer, Michael Zemlin, Johannes Steinhard

**Affiliations:** 1 Hospital for General Pediatrics and Neonatology, Saarland University Medical Center, 66421 Homburg/Saar, Germany; 2 Pränatalmedizin Dorsten, Dorsten, Germany; 3 LARA-Praxis für Frauengesundheit, Bocholt, NRW Germany; 4 Fetal Cardiology, Heart and Diabetes Center NRW, Ruhr-University Bochum, Bad Oeynhausen, Germany; 5 Prenatal Medicine Center Münster, Münster, NRW Germany

**Keywords:** Fetal echocardiography, Ultrasound didactics, Ultrasound simulation training, Simulation-based ultrasound training, Prenatal detection rate, Congenital heart disease

## Abstract

**Purpose:**

To analyze the learning curves of ultrasound novices in fetal echocardiography during structured simulation-based ultrasound training (SIM-UT) including a virtual, randomly moving fetus.

**Methods:**

11 medical students with minimal (< 10 h) prior obstetric ultrasound experience underwent 12 h of structured fetal echocardiography SIM-UT in individual hands-on sessions during a 6-week training program. Their learning progress was assessed with standardized tests after 2, 4, and 6 weeks of SIM-UT. Participants were asked to obtain 11 fetal echocardiography standard planes (in accordance with ISUOG and AHA guidelines) as quickly as possible. All tests were carried out under real life, examination-like conditions on a healthy, randomly moving fetus. Subsequently, we analyzed the rate of correctly obtained images and the total time to completion (TTC). As reference groups, 10 Ob/Gyn physicians (median of 750 previously performed Ob/Gyn scans) and 10 fetal echocardiography experts (median of 15,000 previously performed Ob/Gyn scans) were examined with the same standardized tests.

**Results:**

The students showed a consistent and steady improvement of their ultrasound performance during the training program. After 2 weeks, they were able to obtain > 95% of the standard planes correctly. After 6 weeks, they were significantly faster than the physician group (*p* < 0.001) and no longer significantly slower than the expert group (*p* = 0.944).

**Conclusion:**

SIM-UT is highly effective to learn fetal echocardiography. Regarding the acquisition of the AHA/ISUOG fetal echocardiography standard planes, the students were able to reach the same skill level as the expert group within 6 weeks.

## What does this study add to the clinical work?

**Table:** 

Simulation-based ultrasound training with a randomly moving fetus is highly effective and could be a gamechanger in teaching fetal echocardiography. More studies are needed to examine the transferability of simulation-based skill achievements into clinical settings	

## Introduction

The improvement of neonatal outcomes remains one of the major global health goals. Congenital heart defects (CHD) are the most common birth defects with a prevalence of approximately 1% in all live births [1, 2]. Prenatal Diagnosis (PD) of CHD enables an elective, planned delivery in experienced tertiary centers with standby pediatric cardiology, neonatology and heart surgery teams [3]. Immediate postnatal interventions like balloon atrial septostomy might be necessary. Correct PD secures best outcome in these patients, it has been shown to reduce neonatal morbidity and mortality in complex heart failures like hypoplastic left heart syndrome (HLHS), transposition of the great arteries (TGA) and coarctation of the aorta [4–8]. Furthermore, prenatal therapy such as intrauterine dilation of critical fetal aortic or pulmonary stenosis or atresia can be conducted, which can prevent the progression of the disease into single ventricle circulation [9].

In Germany, only 12% of all CHD and 41% of all severe CHD are diagnosed prenatally [1]. Regardless of improved technical capabilities like high-resolution ultrasound devices, recognition rates remained low within the last decades [10, 11]. CHD detection rates differ largely between tertiary fetal cardiology centers and baseline pregnancy screening providers [12]. As most fetal CHD occur in low-risk pregnancies, only 30.7% of CHD pregnancies underwent fetal echocardiography [13]. Here, multi-level screening systems like the German system are needed to optimize the sensitivity of baseline screening providers to increase prenatal detection rates of CHD [10]. Hence, training fetal echocardiography skills of baseline screening providers is a crucial step and a cornerstone for the improvement of CHD detection rates in existing multi-level screening systems [11]. Various training programs for obstetric ultrasonographers have proven to be effective [14, 15]. Some ultrasound (US) training programs were able to improve the prenatal detection rate of CHD tremendously. Uzun et al. established a nationwide training network in Wales, consequently the perinatal mortality of neonates with CHD decreased significantly. The preoperative mortality of children with TGA dropped to 0%, and survival ofneonates with HLHS improved from 30% to more than 75% [15]. Simulation-based ultrasound training (SIM-UT) approaches may build a new way to train sonographic and diagnostic skills of obstetricional physicians as well as ultrasound novices [16, 17]. Yet, the effectiveness of structured SIM-UT for training in fetal echocardiography has never been analyzed prospectively. In the FESIM II study (FEtal SIMulation), a 6-week trial with the Simbionix US Mentor (Simbionix, Beit Golan, Israel) was conducted to test the feasibility and effectiveness of SIM-UT in fetal echocardiography. We aimed to analyze learning curves of ultrasound novices in an extended fetal echocardiography protocol and compared their performance with two reference groups of (1) obstetric–gynecologic (Ob/Gyn) physicians and (2) fetal echocardiography experts.

## Methods

11 medical students from year 3 to 6 were included as ultrasound beginners. Inclusion criteria were (a) enrollment as a medical student, (b) completion of the subject of anatomy, (c) less than 10 h of experience in Gyn/Ob US. Additionally, as reference groups, 10 Ob/Gyn physicians and 10 fetal echocardiography experts were included separately. All medical students attended an introductory 90-min seminar followed by individual hands-on training sessions with the ultrasound simulator. The two initial training sessions were supervised by student tutors familiar with the simulator who gave a 30-min introduction to each participant. The following training sessions were completed by the participants alone, student tutors were available in case of questions. The medical students trained two times per week and gathered 12 h of individual hands-on training time per person during the training period. Training and tests focused on the correct acquisition of standard planes on a healthy fetus as an essential basis for any sonographic assessment. The trainees were able to train the standard planes on pathologic cases, since the simulator offers virtual fetuses with HLHS, TGA and Tetralogy of Fallot. The progress of the trainees was analyzed in standardized tests every 2 weeks: Participants were asked to obtain and record all 11 correct standard views as quickly as possible on a healthy, randomly moving virtual fetus*.* Tests were carried out under real life examination conditions with all aids being removed. All points in time when participants froze or unfroze an ultrasound image or decided to abort the search for a certain standard plane were recorded.

All members of the reference groups received a questionnaire to quantify their previous US experience. Additionally, they were asked to give information regarding their professional career, as year of medical license or specialization as well as level of qualification according to the German Society of Ultrasound in Medicine (Deutsche Gesellschaft für Ultraschall in der Medizin, DEGUM). The standard planes trained in the study (Table 1) were defined in accordance with the guidelines of the American Heart Association (AHA), the International Society for Ultrasound in Obstetrics and Gynecology (ISUOG) [18], the American Institute of Ultrasound in Medicine (AIUM) [19] and the American Society of Echocardiography (ASE) [20].

**Table 1:** The fetal echo standard planes trained by the students during the study

Transverse standard planes	Sagittal standard planes	Oblique standard planes
Transverse abdominal view (Abd)	Aortic arch view (Ao)	High short axis (HSX, “circle and sausage” view)
Four chamber view (4CV)	Ductal arch view (Duc)	Low short axis (LSX, biventricular view)
Left ventricular outflow tract view (LVOT)	Bicaval view (Bic)	
Right ventricular outflow tract view (RVOT)		
Three vessel view (3V)		
Three vessel trachea view (3V-T)		

All standard planes as proposed in the AHA and ISUOG guidelines

In its examination mode, the US simulator automatically rated the obtained standard views as correct or incorrect after the completion of the examination. During the trial we noticed that the simulator sometimes did rate correct planes as incorrect, hence a second level of examination had to be implemented. If a frozen standard view was rated as incorrect by the simulator, a scientific expert panel consisting of three fetal echocardiography experts, all members of a quality management committee (“*KVWL-Qualitätssicherungskommission*”) and each at least certified as DEGUM level II, was considered to rate the obtained views in a blinded fashion and independently either as correct or incorrect by retrospective video rating. These planes were reevaluated using the grades of the German school system, ranging from “1—very good” to “6—insufficient”. Afterwards, the average grades of all rated standard views were calculated. Standard planes with average grades “1–4” were re-evaluated as correct.

### The ultrasound simulator

**Fig. 1:**
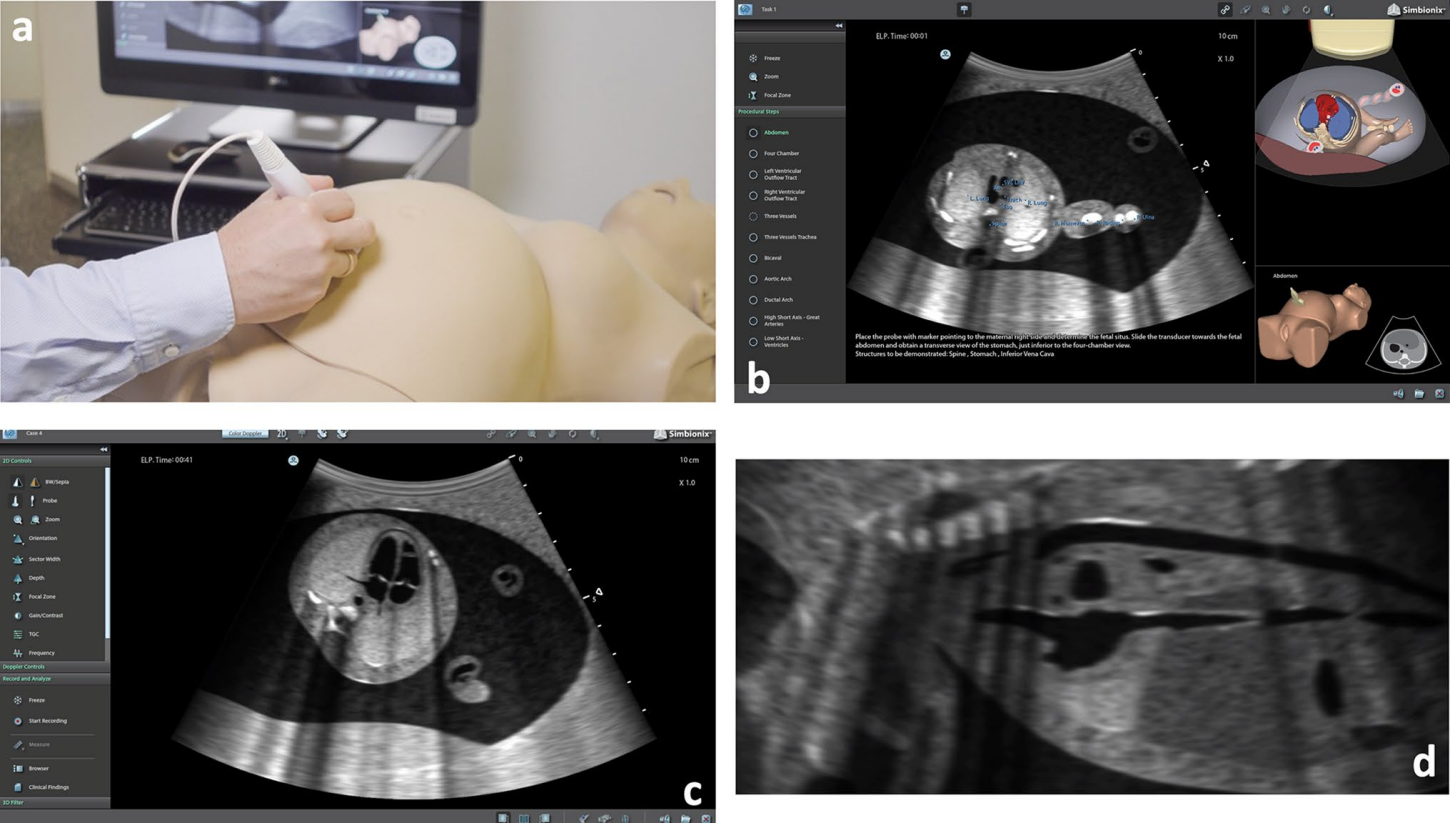
The US Mentor ultrasound simulator: **a** Setup of the simulator. **b** Screen in “Task mode” (with aids), three vessel view. *On the left side*: the desired standard plane can be selected. *Center*: B-mode image including anatomic labels. *Below the B-mode image*: written instructions on how to obtain the correct standard plane. *Right top corner*: 3D model of the fetus demonstrating the current plane. *Right bottom corner*: Position of the probe on the mannequin. **c** Screen during a standardized examination, four chamber view. No aids are given by the simulator. *Left side*: “Knobology” of the ultrasound machine, e.g., adjustment of the depth or focus zone. **d** A bicaval plane assessed as incorrect by the simulator that was later rated as correct (Grade 1−) by the expert panel

Our study was conducted on the 3D Systems Simbionix US Mentor (Simbionix, Beit Golan, Israel) ultrasound simulator consisting of a mannequin, various sham ultrasound probes, a touchscreen with a built-in PC, a footswitch, a wireless mouse, keyboard and the “electronic box” as the central computing device connected to the transmitter (Fig. 1a). The transmitter, a hand-sized box fixated on the back of the mannequin, emits a magnetic field, which is detected by built-in sensors of the sham ultrasound probes. This enables the simulator to locate the exact position of the probe. Subsequently, the simulator renders a real-time image of a simulated fetus by use of a virtual fetus, that was generated using MRI-volume data. The utilization of a virtual fetus enables the simulator to simulate fetal movements while scanning. This feature is unique to the US Mentor. The virtual fetus also facilitates the simulation of various artifacts and enables the implementation of malformations.

### Fetal echo module

The Fetal Echo Module has two different main modes: “Task mode” and “Case mode”. During “Task mode” (Fig. 1b), multiple aids are provided by the simulator: A 3D map of the fetus, anatomic labels on the B-mode image, written instructions on how to obtain the selected standard planes, schematic drawings of standard planes, live visualizations of the probe positioning and live-feedback regarding correctness of the obtained image plane. This creates a learning environment especially suitable for beginners, trainees can familiarize themselves with the standard planes and practice to obtain them.

In “Case mode” (Fig. 1c) a full echocardiographic examination of the fetus including B-mode, M-mode and biometric measurements as well as Doppler ultrasonography including PW-Doppler and adjustment of the pulse repetition frequency can be done. All aids provided by the simulator during “task mode” can be deactivated if the examination is supposed to be conducted under real life-examination conditions. After using “Case mode,” the US simulator will provide an individual report each time the user ends the simulation, including data about how many standard planes were obtained correctly or incorrectly. All obtained images can be viewed again and are compared with a gold standard image.

### Statistical analysis

After the completion of the examinations, time intervals needed by the participants to obtain the correct standard views were calculated. The rate of appropriate images (RAI) was defined as the percentage of correctly obtained standard planes out of all standard planes. Total Time to Completion (TTC) was defined as the timespan needed by participants to obtain all eleven standard planes. Statistical analysis was performed using IBM SPSS Statistics 27. Paired nonparametric tests (Friedman and Wilcoxon) were used to compare RAI and TTC of the student group between their three examinations after 2, 4, and 6 weeks. With nonparametric unpaired tests (Kruskal–Wallis and Mann–Whitney *U*) we compared performance values between students, physicians and DEGUM II level US experts. A *p* < 0.05 was considered as significant.

## Results

### The demographics and composition of the reference groups

The physician group had previously performed a median of 750 Ob/Gyn ultrasound scans. Nine out of ten physicians were working at a large level-1 center with > 2500 births annually. The group consisted of four young Ob/Gyn doctors (PGY-1 and -2), four senior Ob/Gyn residents (PGY-4 and -5), one Ob/Gyn specialist and one Ob/Gyn attending physician.

The expert group consisted of 10 highly specialized fetal medicine experts. All of them were certified at least at DEGUM level II and all of them had previously performed more than 1000 fetal echocardiograms. The median numbers of previously performed examinations were 15,000 Ob/Gyn ultrasound scans, 8000 fetal biometries, 6000 DEGUM level II scans and 2500 fetal echocardiograms.

### Rate of appropriate images (RAI)

After 2 weeks of training, the medical students obtained more than 95% of the standard planes correctly (Table 2). This rate remained steady after 4 and 6 weeks of training. Likewise, the expert group obtained more than 95% of the standard planes correctly. The physician group obtained 92% of the standard planes correctly, the difference to the student group was not significant (*p* < 0.576). All other tests turned out insignificant as well, indicating that the RAIs in all groups are comparable.

**Table 2:** Rate of appropriate images according to groups / training time

Group	Rate of appropriate standard planes (%)
Students after 2 weeks	96.7 ± 7.4
Students after 4 weeks	96.7 ± 4.6
Students after 6 weeks	95.9 ± 1.1
Physician group	91.8 ± 13.2
Expert group	95.5 ± 7.7

### Total time to completion (TTC)

**Fig. 2:**
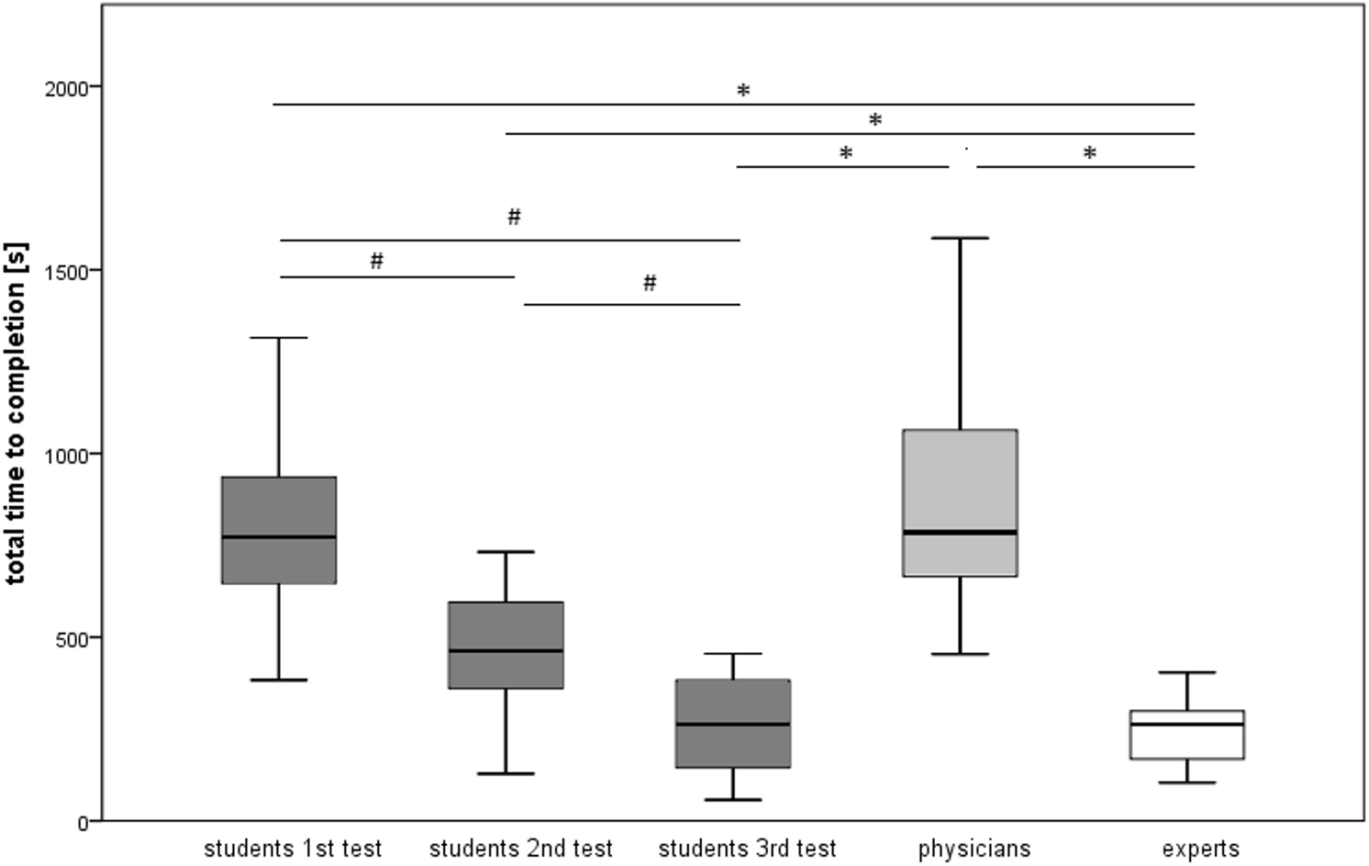
Boxplot showing the distribution of total time to completion (TTC) in seconds of all standard planes by students after 2, 4, and 6 weeks training time compared to physician and expert groups. ^#^Significant difference within groups using Wilcoxon test (*p* < 0.05), *significant difference between groups using Mann–Whitney *U* test (*p* < 0.05)

The medical students showed a steady decrease both in TTC as well as the time to completion of individual standard planes during the study period (Table 3 and Fig. 2). Friedman- and Wilcoxon-tests demonstrate a significant stepwise decrease in the students’ TTC in each test after 2, 4, and 6 weeks (*p* < 0.05).

**Table 3:** Total time to completion (TTC) in seconds according to groups/training time

	*n*	Mean value	Standard deviation	Standard error	95% confidence interval		Minimum	Maximum
					Lower bound	Upper bound		
Students 2 weeks	11	847.09	352.605	106.314	610.21	1083.97	383	1609
Students 4 weeks	11	565.91	442.379	133.382	268.71	863.1	128	1800
Students 6 weeks	11	289.27	221.511	66.788	140.46	438.09	57	825
Physicians	10	951.3	438.826	138.769	637.38	1265.22	454	1800
Experts	10	269.2	128.44	40.616	177.32	361.08	104	543

The Mann–Whitney-*U*-test showed that students became significantly faster than the physician group after 6 weeks (*p* < 0.001) and were no longer significantly slower than the expert group (*p* = 0.944). The physician group was significantly slower than the expert group (*p* < 0.001).

### Time to completion of individual standard planes by group

**Fig. 3:**
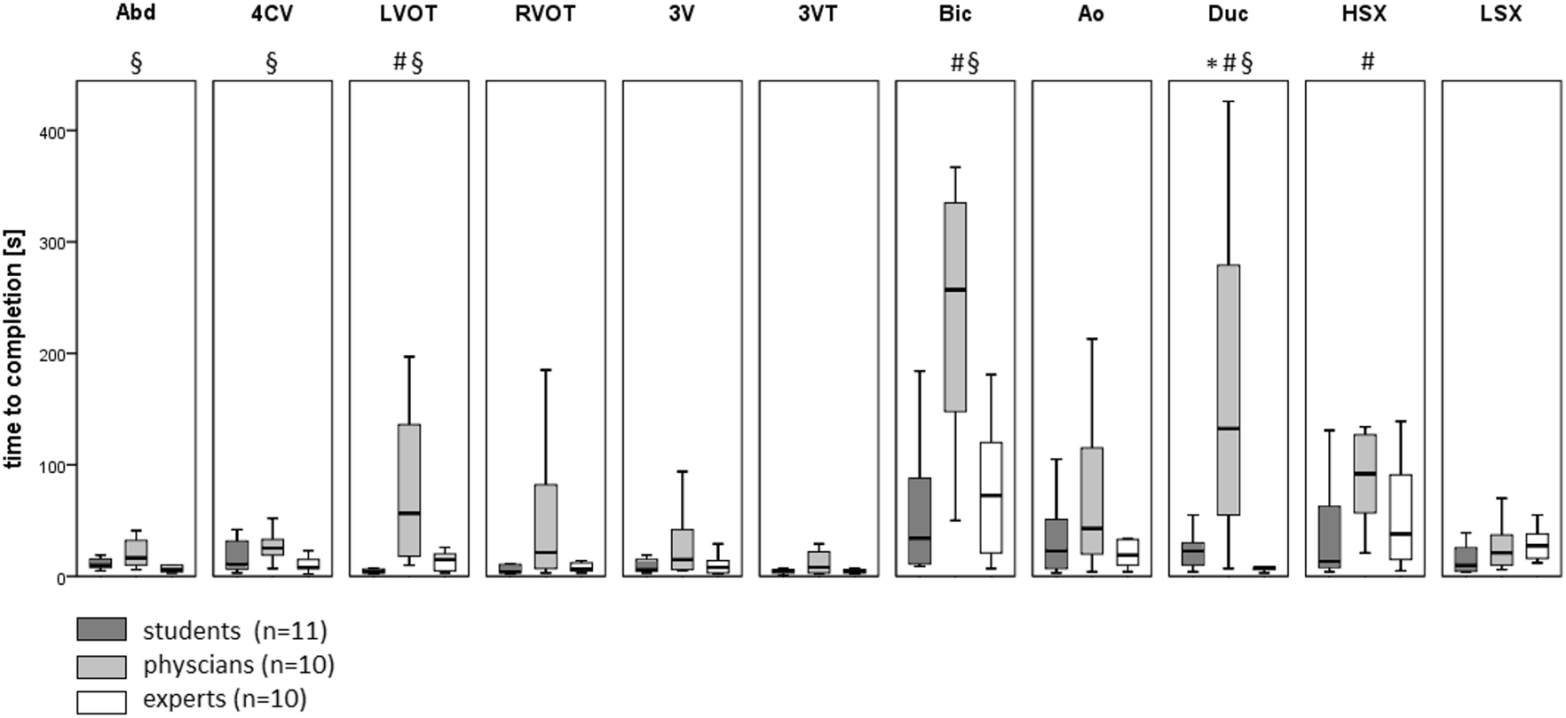
Boxplot showing the distribution of time to completion [s] of single planes between students (after 6 weeks training), physicians and experts. Groups were compared by Mann–Whitney *U*-test. A *p* < 0.05 was considered as significant. Standard views: Abd (transverse abdominal), 4CV (four chamber), LVOT (left ventricular outflow tract), RVOT (right ventricular outflow tract), 3V (three vessels), 3VT (three vessels trachea), Bic (bicaval), Ao (aortic arch), Duc (ductal arch), HSX (high short axis), LSX (low short axis). ^#^Significant difference between students and physicians, *significant difference between students and experts, ^§^significant difference between physicians and experts

Significant (*p* < 0.05) differences in time to completion of individual standard planes between the groups were found in 6 out of 11 standard planes (Fig. 3). The LVOT, bicaval, ductal arch and high short axis views were obtained significantly faster by the students than by the physician group. The expert group obtained the ductal arch view significantly faster than the students and the transverse abdominal, four chamber, LVOT, bicaval and ductal views significantly faster than the physician group. The abdominal transverse, 4CV, 3V, 3VT, and LSX views were obtained by all groups comparatively quickly. In contrast, the time to completion was more heterogeneous for the right/left outflow tract, bicaval, aortic arch, ductal arch and high short axis views.

## Discussion

In this study we could demonstrate that SIM-UT is a highly effective and feasible method to teach fetal echocardiography. Medical students, as ultrasound beginners, managed to obtain more than 95% of all standard planes correctly after a short training period of 2 weeks only. Significant learning progress was achieved within a brief timeframe. Visuo-spatial ability, hand–eye coordination and pattern recognition are the most important skills while learning ultrasound. Novices often struggle with the “mental mapping” between 3D anatomic structures and the 2D ultrasound plane [21]. Fetal echocardiography is particularly difficult to learn due to random fetal movements and the absence of visible anatomical landmarks for the probe positioning. Here, visuo-spatial skills are even more important. Computer-based simulation systems display a promising new opportunity to train these skills. For example, the 3D anatomical map given by the simulator during the training in “Task mode” might be a very useful asset regarding the development of visuo-spatial skills and mental mapping. In addition, this model is the first ultrasound simulation system that encompasses the simulation of random fetal movements and addressing this particular challenge in learning fetal echocardiography. Various levels of difficulty can be simulated. Different simulation settings like Task and Case mode are provided. Trainees can independently switch aids on or off ensuring that basic and advanced sonographers can adjust difficulty of training to their personal skill level. According to our results, Ob/Gyn physicians in Germany could benefit from training with the simulator, as their skill level in fetal echocardiography was comparable to the students’ skill level after 2 weeks of training. The performance of the physician group might be regarded as representative of the level of basic screening skills in Germany. They had performed a median number of 750 Ob/Gyn ultrasound scans, meeting the requirements of the German medical specialist standard (“Facharztstandard”), which allows physicians to practice independently.

The simulator evaluated the obtained standard planes very strictly, standard planes were classified as incorrect despite being clinically correct. To avoid a bias in favor of the student group, who had more previous training time with the simulator than the reference groups and therefore knew the simulators specifications better, an expert panel reevaluated all standard planes classified as incorrect by the simulator.

### Transferability into real ultrasound

Main limitation of our study is the missing clinical transfer. Due to the COVID-pandemic, outpatients were seen under strict hygienic precautions and the student group could not be tested as planned on pregnant women. However, the simulator creates a very realistic simulation environment. Additionally, it has been demonstrated that skill assessment using ultrasound simulators is a reliable and valid method [22, 23], convincing us that our results are also transferable into a clinical setting.

We examined learning curves of medical students and their skill acquisition in obtaining 11 different standard planes in accordance with the ISUOG and AHA guidelines. The acquisition of these standard planes represents a thorough examination of the fetal cardiovascular system, exceeding the requirements for the standard obstetric level of care in Germany and fulfilling the ISUOG and DEGUM guidelines for fetal echocardiography [18, 24, 25].

Another limitation of our study is that the students’ ability to recognize abnormal findings was not tested. Follow-up studies are needed to assess the effectiveness of SIM-UT regarding the detection of pathologic findings. However, mastering the standard planes and the exact knowledge of normal findings of the standard planes is the key and cornerstone to better detection of anomalies. It is important to know the “healthy structures” in standard planes, to thereby train the eye to detect deviations from standard. In this regard, specific training on the simulator is time efficient and learner centered. These basics are otherwise often acquired alongside clinical practice and not given enough emphasis. The importance of the correct acquisition of standard planes is also emphasized by Van Nisselrooij et al., who have demonstrated that incorrectly obtained standard planes are the main reason for missed prenatal CHD diagnoses, accounting for 50% of all missed diagnoses in the Netherlands [26]. They conducted a case–control-study analyzing second trimester screening scans of children born with severe CHD. Further 30% of CHD were missed due to lack of recognition of visible pathologies, with a remaining 20% being missed inevitably with good quality of the images but no visible defects. Since training of the correct acquisition of standard planes is the major focus of the fetal echocardiography module, we conclude that structured SIM-UT has a high potential to increase the prenatal detection rate of CHD.

To our knowledge, this study is the first trial which prospectively gathered and analyzed data on structured SIM-UT in fetal echocardiography. A major strength of the current study is the virtual fetus, creating a real-life situation by moving randomly throughout the examination. This allows the evaluation of ultrasound skills in a real-life setting. In future, ultrasound simulators may be increasingly used as a quality assurance tool. Standardized examination conditions, patient-independent availability, implemented pathologies as well as an assessment automatically provided by the simulator create excellent frameworks for both ultrasound training as well as quality assurance [22, 23]. Another major strength of our study are the two reference groups. Overall, the physician group might be regarded as representative for the standard of care in Germany with the skill level of the expert group being representative for the level of care at highly specialized fetal medicine tertiary center.

### Reproducibility and implementation into curricula

During the study, the students trained independently and alone with the simulator. The simulator’s teaching module provides the possibility to implement teaching videos or supplemental didactic material into the training. This makes autonomous learning more feasible even for difficult and specialized skills like fetal echocardiography. An obstacle in implementing SIM-UT into standard curricula are relatively high acquisition costs. As structured supervision in conventional ultrasound training is time and cost intensive, SIM-UT is an option and proven to save resources over time [27].

An alternative didactic method to teach ultrasound skills are peer-assisted teaching concepts, which have been demonstrated to be effective [28, 29]. Recently, Recker et al. developed a peer-teaching-based comprehensive structured training curriculum for residents learning gynecologic ultrasound [30]. The development and implementation of such training programs for fetal echocardiography might be at least similarly or more effective as SIM-UT. However, our study group speculates that SIM-UT might be superior to conventional live-model-based ultrasound training during the initial training phase where trainees need to familiarize themselves with the sonographic standard planes since standardized examination conditions, patient-independent availability, and the simulators aids such as the 3D map create an ideal learning atmosphere. Nevertheless, it is important to note that peer-teaching-based training approaches and SIM-UT should not be seen as two competing rival methods—simulation-based training can be integrated into peer-teaching training programs. This might lead to synergistic effects and improve the overall learning outcome.

## Conclusions

Structured SIM-UT is feasible and highly effective in learning and teaching fetal echocardiography. Regarding the acquisition of standard planes, students were able to reach the skill level of the expert group within 6 weeks. Ultrasound beginners and Ob/Gyn physicians can both benefit from structured SIM-UT. SIM-UT may have the potential to increase prenatal detection rates of CHD and improve neonatal outcome. Based on our results we recommend more studies to examine transferability of simulation-based skill acquisition into clinical settings.

## Data Availability

The data that support the findings of this study are available from the corresponding author upon reasonable request.

## Abbreviations

SIM-UT: Simulation-based ultrasound training
PD: Prenatal diagnosis
US: Ultrasound
DEGUM: Deutsche Gesellschaft für Ultraschall in der Medizin (German Society for Ultrasound in Medicine)
ISUOG: International Society of Ultrasound in Obstetrics and Gynecology
RAI: Rate of appropriate images
TTC: Total time to completion
PGY: Postgraduate year (equaling the years of clinical experience as a doctor)
